# Utility of a Simplified Iliac Artery Calcium Scoring System to Guide Perioperative Management for Renal Transplantation

**DOI:** 10.3389/fmed.2021.606835

**Published:** 2021-03-16

**Authors:** Evan C. Werlin, Hillary J. Braun, Joy P. Walker, Jonathan E. Freise, Dominic Amara, Iris H. Liu, Anna Mello, Mehdi Tavakol, Peter G. Stock, Jade S. Hiramoto

**Affiliations:** ^1^Department of Surgery, University of California, San Francisco, San Francisco, CA, United States; ^2^Division of Cardiothoracic and Vascular Surgery, Ohio Health Hospital System, Columbus, OH, United States; ^3^School of Medicine, University of California, San Francisco, San Francisco, CA, United States

**Keywords:** kidney, transplantation-kidney, calcification, vascular & endovascular surgery, peripheral arterial disease (MeSH)

## Abstract

Non-contrast computed tomography scans of the abdomen and pelvis (CTAP) are often obtained prior to renal transplant to evaluate the iliac arteries and help guide surgical implantation. The purpose of this study was to describe the association of iliac calcification scores with operative and clinical outcomes using a simplified scoring system. A retrospective review of 204 patients who underwent renal transplant from 1/2013 to 11/2014 and who had a CTAP within 3 years prior to transplant was performed. Data were collected from the electronic medical record. Common iliac artery (CIA) and external iliac artery (EIA) calcification on CTAP were assessed using a simple scoring system. Descriptive statistics, logistic regression, and survival analyses were performed. A total of 204 patients were included in the analysis. The mean age was 57.4 ± 11.2 years and 134/204 (66%) were men. Nineteen patients (9%) had a history of peripheral artery disease (PAD), 78 (38%) had coronary artery disease, and 22 (11%) had a previous cerebrovascular accident (CVA). Patients with severe right EIA plaque morphology were significantly more likely to require arterial reconstruction compared to those without severe plaque (3/14[21%] 4/153 [3%], *p* = 0.03). Eleven patients (5%) had one or more amputations (toe, foot, or transtibial) following transplant. In UV logistic regression, severe EIA plaque morphology (OR 8.1, CI 2.2–29.6, *p* = 0.002) and PAD (OR 10.7, CI 2.8–39.9, *p* = 0.0004) were associated with increased odds of amputation. In the MV model containing both variables, EIA plaque morphology (OR 4.4, CI 0.99–18.3, *p* = 0.04) and PAD (OR 6.3, CI 1.4–26.4, *p* = 0.01) remained independently associated with increased odds of amputation. Over a median follow up of 3.3 years (IQR 2.9–3.6), 21 patients (10%) had post-operative major adverse cardiac events (MACE, defined as myocardial infarction, coronary intervention, or CVA), and 23 patients died (11%). In unadjusted Kaplan Meier analysis, CIA plaque (*p* = 0.00081) and >75% CIA length calcification (*p* = 0.0015) were significantly associated with MACE. Plaque burden in the EIA is associated with increased need for intra-operative arterial reconstruction and post-operative lower extremity amputations, while CIA plaque is associated with post-operative MACE. Assessment of CIA and EIA calcification scores on pre-transplant CT scans in high risk patients may guide operative strategy and perioperative management to improve clinical outcomes.

## Introduction

Peripheral artery disease (PAD) is highly prevalent among patients with chronic kidney disease (CKD) and end stage renal disease (ESRD), impacting anywhere from 7 to 48% of this patient population (1, 2). Symptomatic PAD is associated with an increased risk of delayed graft function (DGF) and renal allograft failure (3), but the majority of patients with ESRD awaiting renal transplantation have asymptomatic PAD (4). These patients commonly have atherosclerosis and arterial calcification affecting the iliac arteries, which may increase the complexity or even preclude the possibility of a successful arterial anastomosis during renal transplantation (5–7). Several studies have shown that the severity of atherosclerosis negatively correlates with patient (8–11) and graft survival (7, 12), and arterial calcifications have been associated with higher rates of post-transplant cardiovascular (CV) morbidity, all-cause mortality (11), and operative complexity (13).

PAD is also associated with an increased risk of cardiovascular (CV) and all-cause mortality (11, 14–16) that persists following renal transplantation (17). While renal transplantation offers a significant survival benefit to patients with ESRD (18), these patients continue to be at high risk for CV complications and death, even with a functioning renal allograft in place (11, 14) given the burden of their medical comorbidities and the additive effects of immunosuppression. Preoperative evaluation of the extent and severity of PAD in high risk patients is therefore important not only for operative planning, but also for management and risk stratification of these patients after transplantation. Furthermore, with the persistent shortage of organ donors and extensive waitlist times, it is imperative that we utilize all available predictive tools to maximize operative success and long-term clinical outcomes.

The severity and extent of vascular calcifications in patients undergoing renal transplantation have been assessed using a variety of scoring systems (9–11, 13, 19–21). Some studies have used pelvic radiographs (20, 21) while others have used computed tomographic (CT) imaging (9–11, 13, 19), however, no standardized guidelines exist for the pre-operative evaluation of vascular calcifications in patients undergoing renal transplantation. An ideal scoring system would be simple to perform, aid in appropriate patient and target artery selection, and contribute to improved technical and graft-related outcomes.

At our institution, renal transplant candidates with long-standing diabetes mellitus (DM) or known PAD undergo a non-contrast CT scan of the abdomen and pelvis (AP) prior to renal transplantation to evaluate the extent of calcific atherosclerotic disease in the target vessels to help plan the operative approach. The purpose of the present study was 3-fold: first, to examine the extent and distribution of common iliac artery (CIA) and external iliac artery (EIA) calcifications; second, to determine the associations of CIA and EIA calcifications with operative complications and clinical outcomes following renal transplantation, and third, to propose a modified and simplified scoring system for expedient pre-operative CIA and EIA evaluation.

## Materials and Methods

### Patient Population and Clinical Outcomes

This study was approved by the Institutional Review Board at the University of California San Francisco (IRB #16-21401). A retrospective chart review was performed between January 2013 and November 2014 to identify all adult patients who underwent renal transplantation, as well as those who had a CT AP within 3 years prior to renal transplant. A CT AP is routinely obtained on all patients that meet at least one of the following criteria: (1) Need for pre-operative cardiac catheterization; (2) Type 1 DM with disease >20 years; (3) Type II DM with disease >10 years; (4) All patients >70 years old; (5) History of PAD. Electronic medical records were reviewed to assess demographic variables, co-morbidities [PAD, hypertension (HTN), hyperlipidemia, DM, prior solid organ transplant, history of smoking, chronic obstructive pulmonary disease, prior stroke, coronary artery disease (CAD), congestive heart failure, prior myocardial infarction, prior percutaneous coronary intervention, prior coronary artery bypass graft], medications (aspirin, statin, beta blocker), operative complications, graft-related outcomes, CV-disease outcomes, and patient survival. Post-operative cardiac events were defined as a myocardial infarction (MI), coronary intervention, or cerebrovascular accident (CVA). Mortality was determined through chart review as well as querying of the Social Security Death Index.

### Image Analysis

All CT scans had a minimum of 5 mm slice thickness and were analyzed in the axial plane of the non-contrast phase for evaluation of vascular calcifications. The CIA was defined as the artery beginning at the inferior aspect of the aortic bifurcation and extending to the bifurcation of the vessel into the EIA and internal iliac artery. The inferior border of the EIA was defined by the superior aspect of the femoral head. Vascular calcifications were graded as none to moderate or severe for morphology, circumference, and length (Table 1) as adapted from a previously described scoring system (13). However, in an effort to create a simplified approach for iliac artery evaluation, we condensed the calcification scores to dichotomous variables. None-moderate plaque was defined as a morphology with ≤2 mm thickness of calcification while severe plaque indicated >2 mm maximal thickness of calcification. None-moderate circumference and length scores were defined as calcifications ≤75% of the circumference and length, respectively. Severe circumference and length scores corresponded to plaque >75% of the circumference or length. All CT scans were reviewed by a second-year vascular surgery fellow blinded to patient demographics and clinical outcomes.

**Table 1:** Simplified dichotomous scoring system for iliac plaque morphology, circumference, and length.

**Morphology/Plaque score**	**Definition**
None-moderate	≤2 mm thickness of calcification
Severe	>2 mm maximal thickness of calcification
**Circumference Score**	
None-moderate	≤75%
Severe	>75%
**Length Score**	
None-moderate	≤75%
Severe	>75%

### Renal Transplant Technique

The choice of recipient artery used for the transplant anastomosis was made by the operating surgeon. Kidneys were routinely implanted into the iliac fossa with the vascular anastomoses to the external iliac vessels as a first choice. In the presence of atherosclerosis of the EIA, presence of a prior allograft or unsuitable anatomy of the EIA, the CIA was used. Laterality of implantation (right or left) was determined based on the quality of the artery, existence of a prior renal allograft, symptomatic claudication in the lower extremity, or other potential complicating factors such as the presence of a right-sided ileostomy. A standard retroperitoneal approach was used for the majority of transplants with the exceptions being for those transplants that were planned pre-operatively to be done to the CIA or abdominal aorta. In these cases, a midline intraperitoneal approach was used. The ureter was implanted into the urinary bladder with a standard ureteroneocystostomy. The use of ureteral stents was determined by the surgeon intraoperatively.

### Statistical Analysis

Statistical analysis was performed with R (version 3.6.2). Measured values are reported as percentages or mean ± standard deviation. The Fisher exact test was used to compare categorical variables, and *t*-tests were used to compare continuous values.

Univariate (UV) and multivariable (MV) logistic regression models were used to determine (a) the association of patient factors with calcification scores, and (b) factors associated with amputation following transplantation. For the outcome of calcification scores, the following variables were evaluated in a UV regression: age, gender, HTN, DM, PAD, CAD, hyperlipidemia, previous CVA, COPD, smoking history, type of renal replacement; variables were included in the MV model if they met statistical significance (*p* < 0.05) in UV analysis. Variables included in the UV analysis of amputation were as follows: age, gender, common iliac calcification scores, external iliac calcification scores, DM, CAD, PAD, type of dialysis, and smoking history. Variables were included in the MV analysis if they met statistical significance (*p* < 0.05) in the UV analysis.

Time to event analyses were performed using the Kaplan-Meier method. Outcomes were graft survival, patient survival, and major adverse cardiac event (MACE, defined as myocardial infarction, coronary intervention, or CVA). A Cox proportional hazards model was also used to identify variables associated with MACE. *P* < 0.05 were considered statistically significant.

## Results

### Patient Characteristics

During the study period, 697 adult patients underwent renal transplantation at our institution. Of these patients, 204 (29%) underwent evaluation with a CT AP within 3 years prior to their transplant. The demographics of this cohort are displayed in Table 2. The mean age for this cohort was 57.4 ± 11.2 years and 66% were men. The majority (191/204, 94%) had hypertension and DM (125/204, 61%), and 78/204 (38%) had coronary artery disease (CAD). 19/204 (9.3%) of patients had a history of PAD and 5/204 (2.5%) had a lower extremity vascular procedure prior to transplant. 190/204 (93%) were on dialysis pre-operatively with 155 (76%) on hemodialysis (HD). 137/204 (67%) received cadaveric renal transplants and 67/204 (33%) received living-donor renal transplants. 61/204 (30%) developed DGF following transplantation.

**Table 2:** Cohort demographics (*n* = 204).

**Demographics**	**Entire cohort**
	**(*n* = 204)**
Age (mean)	57.4
Sex (%male)	134 (65.7%)
Hemodialysis	155 (76%)
PAD	19 (9%)
HTN	191 (93.6%)
HLD	128 (62.7%)
DM	125 (61.3%)
CAD	78 (38.2%)
Prior CVA	22 (10.8%)

### Operative Course

Seven patients required arterial reconstruction during transplantation and all of these transplants were anastomosed to the right EIA. Six patients required tacking sutures to be placed through the iliac artery due to a dissection noted at the time of implantation. One patient lost femoral and pedal pulses at the completion of the transplant and was found to have an extensive dissection of the right EIA that required endarterectomy and placement of an 8 mm Dacron interposition graft. This patient had severe plaque morphology which was circumferential and affected nearly the entire length of the bilateral common and external iliac arteries. Patients with severe right EIA plaque morphology were significantly more likely to require arterial reconstruction compared to those without severe plaque (3/14[21%] vs. 4/153 [3%], *p* = 0.03).

### Calcification Scores

There was no significant difference in calcification scores between the left and right sides. Table 3 shows the distribution of patients with severe plaque morphology, circumference, and length for both the right CIA and EIA. The plaque morphology, circumference, and length were all more extensive in the CIA compared with the EIA. Because the right-sided vessels were more commonly used for the graft anastomoses, all subsequent analyses incorporate use of the right-sided calcification scores.

**Table 3:** Right sided calcification scores (*n* = 204).

**Variable**	**Yes**	**No**
RCIA severe morphology	114 (56%)	90 (44%)
RCIA severe circumference	55 (27%)	149 (73%)
RCIA severe length	108 (53%)	96 (47%)
REIA severe morphology	23 (11%)	181 (89%)
REI severe circumference	31 (15%)	173 (89%)
REI severe length	31 (15%)	173 (85%)

#### RCIA

In MV logistic regression examining factors associated with severe RCIA plaque morphology, and including age, DM, CAD, previous CVA, and smoking (all *p* < 0.05 in UV analysis), recipient age (OR 1.06, CI 1.03–1.1, *p* < 0.001) and CAD (OR 2.4, CI 1.2–4.7, *p*-value 0.01) remained significantly associated with severe RCIA plaque morphology. In a MV logistic regression model including age, gender, CAD, and smoking (all *p* < 0.05 in UV analysis), male gender (OR 2.89, CI 1.01–8.24, *p* = 0.05), CAD (OR 2.53, CI1.08–5.92, *p* = 0.03), and smoking history (OR 1.00–6.03, *p* = 0.05) remained significantly associated with severe RCIA plaque circumference. In a MV logistic regression model including age, PAD, CAD, smoking, and pre-operative hemodialysis (all *p* < 0.05 in UV analysis), older age (OR 1.08, CI 1.04–1.12, *p* < 0.001), PAD (OR 4.00, CI 1.13–14.18, *p* = 0.03), smoking (OR 2.00, CI 1.05–3.81, *p* = 0.03), and pre-operative hemodialysis (vs. peritoneal dialysis) (OR 2.65, CI 1.19–5.90, *p* = 0.02) remained significantly associated with severe RCIA plaque length.

#### REIA

In MV logistic regression examining factors associated with severe REIA plaque morphology, and including both CAD and PAD, only PAD remained significantly associated with severe REIA plaque morphology (OR 8.3, CI 2.8–24, *p* < 0.001). In MV logistic regression model, both PAD (OR 4.38, CI 1.46–13.18, *p* = 0.009) and previous CVA (OR 3.54, CI 1.20–10.40, *p* = 0.02), were significantly associated with severe REIA plaque circumference. For severe REIA plaque length, PAD was the only risk factor significantly associated in UV analysis (OR 6.65, CI 2.15–20.61, *p* = 0.001).

### Impact of Calcification on Post-operative Outcomes

#### Amputations

Eleven patients (5%) had one or more amputations (toe, foot, or transtibial) following transplant. Four of the first amputations occurred on the side ipsilateral to the transplanted kidney. Table 4 shows detailed information regarding these amputations. In UV logistic regression, severe EIA plaque morphology (OR 8.1, CI 2.2–29.6, *p* = 0.002) and PAD (OR 10.7, CI 2.8–39.9, *p* = 0.0004) were associated with increased odds of amputation. In the MV model containing both variables, EIA plaque morphology (OR 4.4, CI 0.99–18.3, *p* = 0.04) and PAD (OR 6.3, CI 1.4–26.4, *p* = 0.01) remained independently associated with increased odds of amputation.

**Table 4:** Breakdown of lower extremity amputation events following transplantation.

**Patient**	**Gender**	**Pre-operative history of PAD**	**Laterality of kidney transplant**	**First amputation type**	**Laterality of first amputation**	**Second amputation type**	**Laterality of second amputation**	**MACE**
1	Male	No	R	Toe	L	Transmetatarsal	R	Yes
2	Female	Yes	R	Toe	L	–	–	No
3	Male	No	R	Toe	L	–	–	No
4	Male	Yes	R	Toe	L	Below knee	L	No
5	Female	Yes	R	Transmetatarsal	L	Transmetatarsal	R	Yes
6	Female	Yes	R	Toe	R	Below knee	R	No
7	Male	No	R	Toe	L	Toe	L	No
8	Male	No	R	Toe	R	Transmetatarsal	R	No
9	Female	Yes	R	Toe	L	–	–	No
10	Male	No	L	Transmetatarsal	L	Below knee	L	No
11	Female	No	L	Toe	L	–		No

#### Post-transplant Cardiac Events

**Figure 1:**
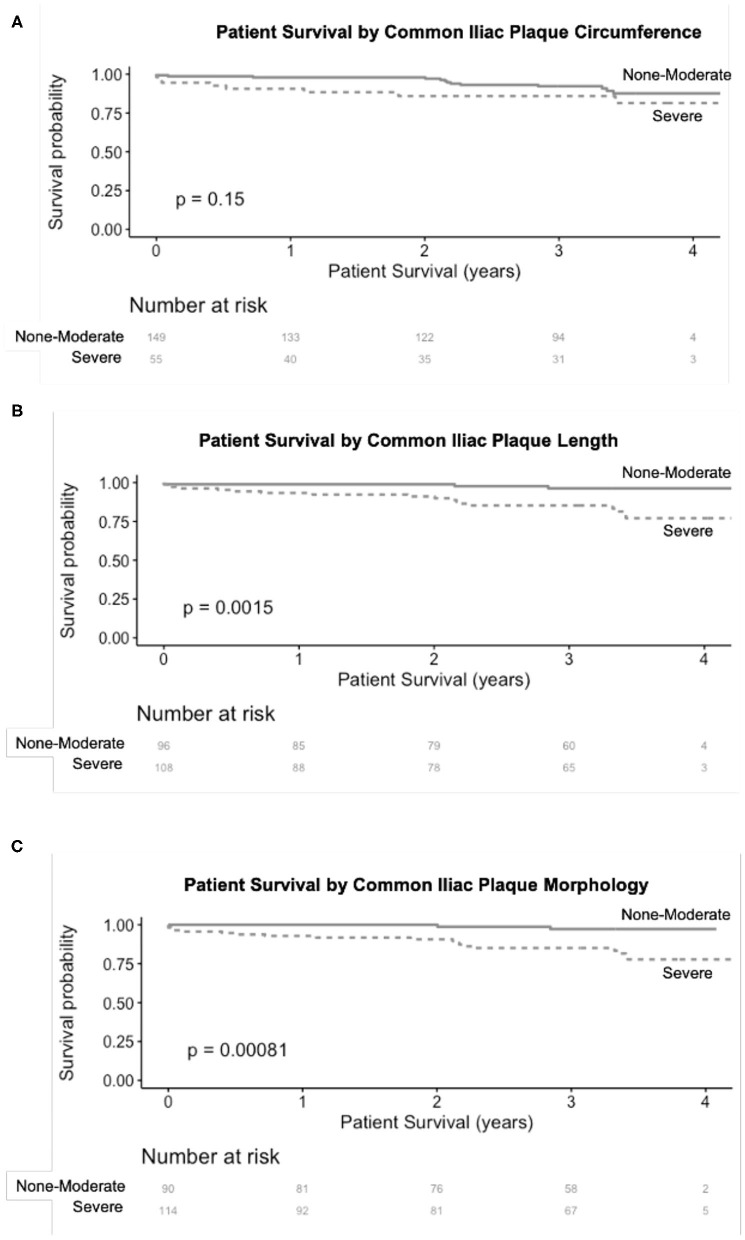
Association of severe CIA plaque circumference **(A)**, length **(B)**, and morphology **(C)** with MACE following renal transplantation. CIA plaque length and morphology both had a significant association with MACE after renal transplantation.

Over a median follow up of 3.3 years (IQR 2.9–3.6), 21 patients (10%) had post-operative MACE and 23 patients died (11%). In unadjusted Kaplan Meier analysis, severe CIA plaque morphology (*p* = 0.00081) and severe CIA plaque length (*p* = 0.0015) were significantly associated with MACE (Figure 1). In a multivariable Cox model, CAD, and CIA morphology remained significantly associated with increased risk of MACE (HR 3.0, *p* = 0.03 and HR 5.8, *p* = 0.02, respectively).

#### Graft and Patient Survival

**Figure 2:**
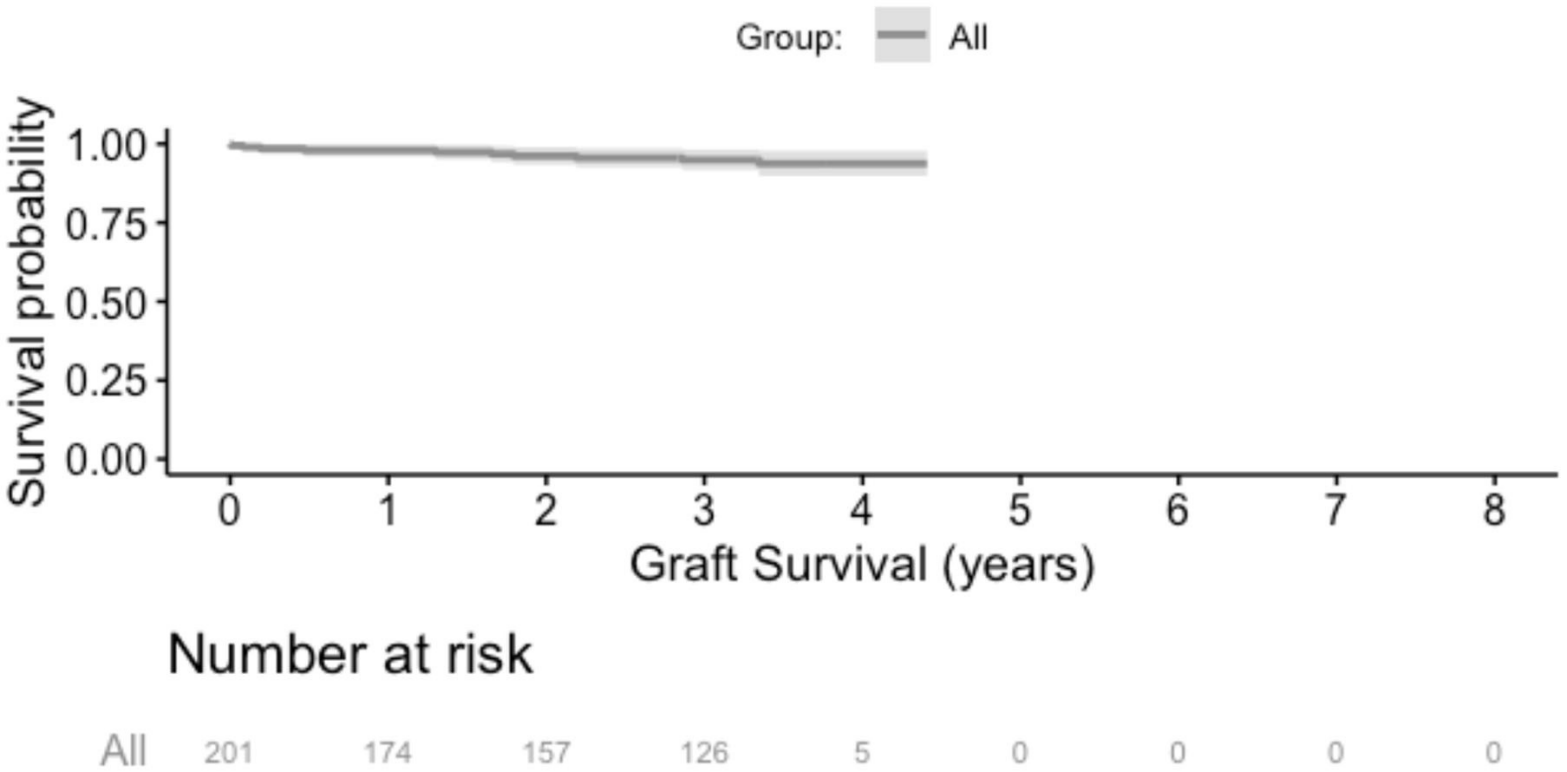
Overall graft survival. There was no difference in graft survival when the analysis was stratified by individual components of the right common or external iliac calcification scores.

**Figure 3:**
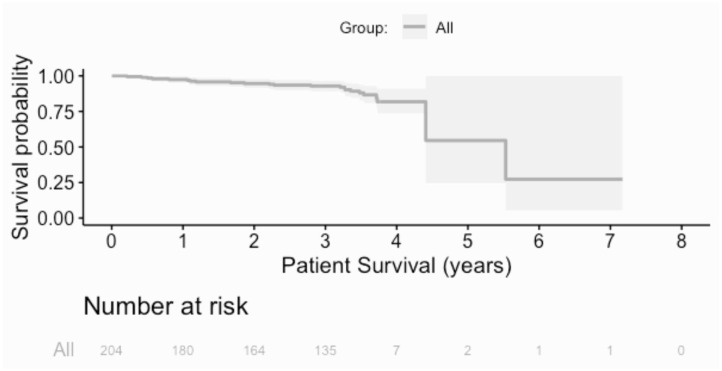
Overall patient survival. There was no difference in patient survival when the analysis was stratified by individual components of the right common or external iliac calcification scores.

Over the follow up time frame, 10 patients (5%) experienced graft failure, defined as retransplantation or return to dialysis (Figure 2). There was no difference in graft survival when patients were by stratified any of the calcification scores for either the RCIA or REIA (all log rank *p* > 0.05). Twenty-three patients (11%) expired during the follow-up period (Figure 3). There was no difference in patient survival when patients were stratified by RCIA or REIA calcification scores (all log rank *p* > 0.05).

## Discussion

This study utilized a simplified CT-based scoring system to classify vascular calcifications in renal transplant recipients and identified associations with iliac artery plaque burden and patient outcomes. The significant findings of this study were 2-fold: (1) more severe EIA calcifications were associated with increased need for intra-operative arterial reconstruction and post-operative lower extremity amputations; (2) Increased CIA plaque burden was associated with post-transplant MACE. These results are clinically significant because the relationship between vascular calcifications and CV morbidity and mortality has been well-described in patients with ESRD and CKD (8, 11, 21), but is less clear in patients who have undergone renal transplantation (7, 10, 11).

No consensus exists on how best to evaluate vascular calcifications identified on pre-operative imaging. Prior studies have demonstrated that plain-film radiographs lack sufficient sensitivity to identify calcifications and subsequently predict post-operative cardiovascular morbidity or mortality (20, 21). CT scans, in contrast, are viewed as a more efficient means of identifying arterial calcifications (19), but still have some limitations. A variety of scoring systems have been applied to pre-operative CT scans in patients undergoing renal transplantation (9–11, 13, 19), and the present study built upon a scoring system proposed by Davis et al. (13) to create a simpler version of the scoring system by dichotomizing the morphology, circumference, and length scores, and still found results similar to prior studies. This modified scoring system allows for a quick and straightforward pre-operative assessment of the calcification scores that can provide important information in planning the operative approach. For example, even though most surgeons prefer the right EIA as the first choice for placement of a renal allograft, if there are areas of >2 mm thickness of calcium, perhaps consideration should be given to the contralateral EIA or either CIA.

Not surprisingly, the rate of CV disease in this study was high, with 38% having a diagnosis of CAD, 11% with history of CVA, and 9% with diagnosed PAD at the time of transplant. We found older age and CAD to be associated with severe plaque morphology in the CIA, but not in the EIA. The distribution of the plaque is important to note since severe plaque in the CIA seems to reflect a higher burden of overall atherosclerotic disease and was associated with a higher risk of post-operative MACE. Severe vascular calcification in the CIA could be considered another CV disease risk factor, and incorporating this into a possible scoring system might be useful in choosing patients to undergo transplantation. In contrast, severe plaque in the EIA was not reflective of underlying CV risk factors in these patients, but played a role in intra-operative technical complications and post-operative amputations.

At our institution, the utilization of pre-transplant cross sectional imaging to evaluate the iliac vessels in kidney transplant candidates with established risk factors for atherosclerotic disease has been of paramount importance in guiding operative planning. In addition to identifying the safest side/site for allograft implantation, the imaging has also prompted more intensive vascular assessment prior to activation for transplantation in patients who were otherwise thought to have clinically silent disease, and visualizing the plaque distribution has enabled us to anticipate potential perioperative complications that may require collaboration with our vascular surgery colleagues. Based on the findings of the present investigation, we do not necessarily feel that we should change our algorithm in determining which patients should undergo a CT scan prior to transplant. Instead, we do think we should change how we further evaluate and care for those patients in whom severe EIA plaque is identified. Patients with severe EIA plaque morphology on pre-operative CT scan are now referred to a vascular specialist for further evaluation, which includes bilateral lower extremity arterial ultrasound examinations and measurements of ankle and toe pressures. It is our hope that a more rigorous pre-operative evaluation in this subset of high-risk patients will be helpful in better determining candidacy for transplantation as well as peri-operative strategies for optimizing outcomes.

This study has several limitations. The results presented here are from a single center with a protocolized system of renal transplant evaluation and therefore may not be generalizable to other centers. This was a retrospective analysis, with the potential for bias from unmeasured confounding variables. The choice of vessel for graft implantation was made by each individual surgeon. We do not know the impact of the pre-operative CT scan on affecting a change in the surgical approach, nor do we know how many patients were screened with a CT scan and had prohibitive iliac calcific disease to proceed with transplantation. Also, only patients who were at high risk for PAD underwent a pre-transplant CT scan, which reduced the overall sample size and our power to detect associations between iliac artery calcifications and graft/patient survival. We also do not have comparative data on outcomes for patients who did not undergo pre-operative CT scans during this period of time. In addition, although most of these patients underwent a pre-operative CT scan based solely on their PAD risk factors, we included all patients who had a CT scan within 3 years of the renal transplant; because of the retrospective nature of this analysis, we are unable to distinguish between these two groups of patients. Finally, our mean study follow up time was ~3 years, so the long term implications of significant plaque burden at the time of kidney transplantation remain unknown.

In summary, local calcified plaque of the recipient EIA is associated with increased operative complexity and higher rates of post-transplant amputation. Plaque burden in the CIA is associated with both patient demographic factors and post-operative MACE and is likely indicative of a greater severity of systemic atherosclerotic disease. Routine pre-transplant CT scans in high risk patients and assessment of the CIA and EIA using a simplified calcium scoring system may guide operative strategy and facilitate perioperative management to improve clinical outcomes.

## Data Availability Statement

The raw data supporting the conclusions of this article will be made available by the authors, without undue reservation.

## Ethics Statement

The studies involving human participants were reviewed and approved by University of California San Francisco IRB. Written informed consent for participation was not required for this study in accordance with the national legislation and the institutional requirements.

## Author Contributions

EW and JH: research design, performance of research, writing of paper, and data analysis. HB: data analysis and writing of paper. JW, JF, AM, and MT: research design and performance of research. PS: research design and writing of paper. IL and DA manuscript writing and revision. All authors contributed to the article and approved the submitted version.

## Conflict of Interest

The authors declare that the research was conducted in the absence of any commercial or financial relationships that could be construed as a potential conflict of interest.
